# Estimated Sweetness in US Diet Among Children and Adults Declined From 2001 to 2018: A Serial Cross-Sectional Surveillance Study Using NHANES 2001–2018

**DOI:** 10.3389/fnut.2021.777857

**Published:** 2021-12-17

**Authors:** Alison Kamil, Alissa R. Wilson, Colin D. Rehm

**Affiliations:** ^1^Health & Nutrition Sciences, Life Sciences, PepsiCo R&D, Chicago, IL, United States; ^2^Health & Nutrition Sciences, Life Sciences, PepsiCo R&D, Purchase, NY, United States

**Keywords:** sugar-sweetened beverages, artificially sweetened beverages, nutrition surveys, cross-sectional studies, trends, United States, National Health and Nutrition Examination Survey, non-nutritive sweeteners

## Abstract

An agreed-upon measure of total dietary sweetness is lacking hindering assessments of population-level patterns and trends in dietary sweetness. This cross-sectional study used 24-h dietary recall data for 74,461 participants aged ≥ 2 y from nine cycles (2001–2018) of the National Health and Nutrition Examination Survey (NHANES) to evaluate trends in the sweetness of the diet in the United States (US). LCS-containing items were matched to a sugar-sweetened counterpart (e.g., diet cola–regular cola or sucralose sugar). The matched pair was used to estimate the sugar equivalents from LCS-sweetened foods or beverages to estimate dietary level sweetness, which was described as grams of approximate sugar equivalent (ASE) per day. Trends in ASE were estimated overall and by subgroup, and trends were further disaggregated by food or beverage category. Overall, LCS sources contributed about 10.5% of ASE. Total ASE declined from 152 g/d to 117 g/d from 2001–2002 to 2017–2018 (*p*-trend < 0.001), with comparable declines in children and adults. Declines in total ASE were predominantly driven by beverages (−36.7% from 2001–2002 to 2017–2018) and tabletop sweeteners (−23.8%), but not food (−1.5%). Observed trends were robust to sensitivity analyses incorporating random, systematic, and sensory trial informed estimates of sweetness and also an analysis excluding possible under-reporters of dietary energy. This practical approach and underlying data may help researchers to apply the technique to other dietary studies to further these questions.

## Introduction

There is a clear, evidence-based global and national public health mandate to limit the consumption of free or added sugars in the diet. The World Health Organization (WHO) currently recommends that the intake of free sugars (defined as monosaccharides and disaccharides added to foods, plus sugars that are naturally present in honey, syrups, and fruit juices) be reduced to less than 10% of total energy intake (1). Additionally, the Dietary Guidelines for Americans 2020–2025 recommends that consumption of added sugars be limited to 10% of calories (2). These recommendations are based on associations between sugar consumption and increased risk of dental caries and on evidence that the consumption of excess energy intake, especially from sugar-sweetened beverages, is associated with weight gain in observational studies (1–3). Because reducing sugar consumption is challenging, some organizations have suggested reducing the consumption of all sweet-tasting foods and beverages, regardless of the source of the sweet taste [i.e., caloric or low- or no calorie sweeteners (LCS)], due to the assumed correlation between dietary sweetness and sugar intake (2, 4, 5).

The scientific evidence to support guidance limiting the sweetness of the total diet is lacking. These recommendations arise from the apprehension that human's innate liking of sweetness (i.e., detected from the sugars naturally present in many foods or added to foods with or without calories) may predispose the development of unhealthy eating behaviors (6). It is hypothesized that increased contact with sweet-tasting foods and beverages could condition palates to desire sweet, increasing liking for sweet taste, and increasing consumption of sweet foods, which may increase risk of developing obesity or metabolic dysfunction. Similarly, this hypothesis generally contends that consumer palates could acclimate to a lower level of sweetness if presented and therefore reduce energy and sugar intakes, supporting weight management (6). Research is needed to test these hypotheses related to the relationship between sweet taste and health.

A generally agreed-upon and validated measure of total dietary sweetness is lacking. Although well-established sensory evaluation techniques in laboratory settings exist for individual foods, agreement on the optimal approach for measuring the sweetness of the total diet is missing, particularly for large-scale population-based studies (6). One of the methods used in research on the effects of sweetness has relied on measures of sweetness intensity for individual food items using trained sensory panelists to develop an estimate of sweetness which is then aggregated into a taste database (7–10). These approaches are useful for evaluating the sweetness of diets on a small population level (e.g., the Dutch diet) but can be resource-intensive and not feasible in regions where there is considerable heterogeneity in the sources of sweetness in the diet.

In addition to the limitations in measuring sweetness of the total diet, there are shortcomings in the assessment of sweetness coming from LCS. While producers and manufacturers are required to list LCS on product ingredients lists, the FDA and other global regulatory authorities do not require disclosure of quantities of LCS in a given product. While trends in consumption of products containing LCS in the United States (US) have been evaluated (11, 12), a methodology is needed to more accurately quantify the contribution of LCS to the sweetness of the total diet. This two-part study aimed to (1) develop a pragmatic methodology for estimating sweetness of the diet that can be used in dietary surveillance and (2) evaluate trends in the sweetness (caloric or LCS) of the diet in US children and adults over the recent decades overall and stratified by food and beverage sources. To address these aims, we used data from nearly 75,000 participants from nine consecutive two-year cycles of the National Health and Nutrition Examination Survey, the cornerstone nutrition surveillance system in the US.

## Materials and Methods

### Data Sources

Data for this project came from nine consecutive cycles of the US National Health and Nutrition Examination Survey (NHANES) from 2001–2002 to 2017–2018. NHANES is a nationally representative survey conducted in two-year cycles that includes the collection of detailed dietary intake data as part of a 24-h dietary recall. Data from the first 24-h recall which is conducted in-person at the mobile examination survey by trained interviewers were used. Details on the 24-h recall are available on the Centers for Disease Control and Prevention (CDC) website, but briefly, trained interviewers collected all foods or beverages consumed in the prior 24-h period from midnight-to-midnight of the prior day (13). Portion-size guides and pictures are available during the dietary interviews, which utilize a multipass method and additional queries for commonly omitted items. A single 24-h dietary recall is appropriate for estimating population-level mean intakes and can be used to estimate trends in dietary intakes (14). Individuals aged ≥ 2 y completing a valid 24-h recall were included in the study. Individuals consuming breastmilk were excluded. Though pregnancy and breastfeeding can impact dietary intakes and preferences, pregnant and lactating women were included in the study to reflect total exposure to sweetness for the entire population. The National Center for Health Statistics (NCHS) obtained Institutional Review Board approval and informed consent was obtained from all subjects; the data have subsequently been made freely available for public use (15).

### Estimating Sweetness of Foods and Diets

To estimate sweetness, we queried the individual foods file that lists all foods or beverages consumed (*n* = 11,192 unique foods) by NHANES participants as part of their 24-h dietary recall. Consistent with prior research, we searched the database for foods that were identified as being “low-sugar,” “diet,” “reduced sugar,” “sugar-free,” or sweetened with “LCS,” etc. (16, 17). The total sugar and added sugar values were inspected, and in some cases, we reviewed ingredient lists for representative products when we were unsure as to whether they contained LCS. Two-hundred and twelve items were identified as meeting these criteria. For each of these items, we then identified the most comparable food or beverage that did not contain LCS. For example, for diet cola and reduced calorie pancake syrup, regular cola and regular pancake syrup were identified as the matched pair. These matched pairs were then used to infer the approximate sweetness of the LCS foods on a gram-per-gram basis. For tabletop sweeteners, we used information from the package regarding equivalency as a gram-per-gram comparison would not be appropriate. For items sweetened with both LCS and traditional caloric sweeteners the matched pair approach was also used (e.g., the matched pair for reduced sugar cola was regular cola). This methodology assumes that the matched pairs were approximately equal in “sweetness,” which was generally corroborated, particularly for beverages and tabletop sweeteners, in a small-scale sensory study conducted prior to initiating this analysis (see Supplementary Materials). The sensory study was used to inform additional analyses undertaken to test the sensitivity of our trend analyses to these assumptions (described below in more detail).

The identification of LCS-sweetened foods and the identification of matches were done by two authors (AK and CDR). The most commonly consumed LCS foods or beverages were overwhelmingly diet beverages (e.g., soft drinks, fruit drinks, teas, and energy drinks) and tabletop sweeteners, and the most common foods were yogurt, gelatin dessert, gum, and pancake syrups. The reliability of the matching process was evaluated and found to have an agreement of 99.4 and 90% in frequency weighted and unweighted analyses. Kappa or chance-corrected agreement was 0.993 and 0.898, suggesting excellent agreement between the two authors. The weighted analyses had an extremely high level of agreement due to the small number of LCS foods that drive the category (e.g., diet soft drinks and individual LCS). Coding discrepancies were discussed and changes were made where appropriate, though most coding discrepancies related to different choices of flavor (e.g., chocolate vs. fruit flavored) where the two choices did not have different sugar content values and would not be expected to change the estimate of sweetness. The matching was done on a cycle-by-cycle basis to account for potential changes in sugar content reflecting updates to the Food and Nutrient Database for Dietary Studies (FNDDS) which are made every new year due to new measurements or potential product reformulation. The individual foods data that report each food or beverage consumed by participants and the amount was used to estimate the total sweetness of each individual 24-h recall. The data were parameterized on a sugar equivalents g/d basis and are referred to as approximate sugar equivalents (ASE) hereafter. The ASE is the sum of total sugar and the estimated sugar equivalents from LCS-containing products.

Because of potential uncertainty around the estimation of the ASE value, multiple sensitivity analyses were incorporated that introduced potential random and systematic error in relative sweetness: random error +/– 20%, random overestimate (0–50%), random underestimate (−50–0%) and a sensory trial informed sensitivity analysis. A uniform distribution was used for the sensitivity analyses introducing random error. The sensory data were not used for the primary analysis as it was based on a small number of highly trained participants and may not reflect the experience of the general population. Details on the sensory study are provided in the Supplementary Table 1. A product-specific constant was applied for foods where the sweetness as reported by trained panelists for the LCS-sweetened version was compared with its counterpart (e.g., diet cola vs. regular cola). For example, sugar and sucralose had identical results in the sensory trial so there was no change in the sensitivity analysis, while diet cola was about 9% less sweet than regular cola and was thus adjusted by this amount. The sensory trial included the most frequently consumed LCS foods (21 products) and categories were applied broadly and not on a flavor-by-flavor or brand-by-brand basis, though there was effort to identify representative foods across major brands. To determine if observed trends were sensitive to exclusion of potential energy under-reporters we conducted an additional sensitivity analysis excluding potential under-reporters following the approach outlined by Murakami and Livingstone (19). A final sensitivity analysis was conducted to address concerns that foods with LCS may be more difficult to identify than beverages/tabletop sweeteners and that consumption of such foods may have increased over the study period. Briefly, we identified the common food categories containing LCS identified by Dunford et al. and systematically imposed a linear trend in the proportion of foods that may have contained LCS from 1% in 2001–2002 to 10% in 2017–2018 and scaling this proportion linearly across this period (20). We then used a uniform distribution to increase the approximate sugar equivalents of these foods by between 10–20%. The food groups evaluated included yogurt, dairy-based desserts, grain-based desserts, candy, other dairy items (e.g., whipped cream and creamers), bars, bread and bread products, sauces and canned/jarred fruit.

### Analysis Approach

After estimating the ASE for each 24-h recall, we estimated the population mean total ASE, and also total sugar and ASE from LCS sources, overall and by population subgroups including by age (2–9 y, 10–19 y, 20–29 y, 30–39 y, 40–49 y, 50–59 y, 60–69 y, ≥ 70 y), gender, race or ethnicity (non-Hispanic white, non-Hispanic black, Mexican-American, other Hispanic, other or mixed race), family income-to-poverty ratio (<1.0 [lower income], 1–1.99, 2–3.99, and ≥ 4.0 [higher income]), education (adults aged ≥ 25 y only: < HS, HS, some college, college graduate+), and body mass index (BMI). The family income-to-poverty ratio is the ratio of family income to the federal poverty guidelines defined by the US Department of Health and Human Services. In 2018, for a family of four living in the contiguous US, the federal poverty guideline was $25,100 (18). BMI categories were defined separately for children or adolescents and adults. For adults, the standard cut points were used for adults, and the age- or sex-specific percentiles from the CDC growth charts were used for children or adolescents. An energy-adjusted measure of total ASE per 2,000 calories was also estimated to compare population subgroups with differing energy requirements.

The survey-weighted mean total ASE, total sugar, and ASE from LCS sources were calculated for each two-year survey cycle, and the statistical significance of trends was evaluated by including survey cycle (e.g., year) in a survey-weighted linear regression model with the sweetness variable of interest as the dependent variable. Primary analyses focused on trends in the total population with secondary analyses examining trends by age (e.g., children or adolescents vs. adults). Analyses also separated ASE from beverages, foods, and tabletop sweeteners and tested for trends within these categories. The average annual change was estimated based on the linear model for the purposes of comparison and for assessing the results of the multiple sensitivity analyses. Trends in ASE were further estimated for eight food or beverage categories to determine whether changes were consistent across categories. These categories included carbonated soft drinks (CSDs), 100% fruit juice, fruit drinks, other beverages, sweets or desserts, fruit, other foods, and sweeteners. Sweets or desserts were defined as sweet-baked goods, jellies, gelatin, ices or popsicles, gum, candy, and milk-based desserts (e.g., ice cream). Sweeteners included tabletop sweeteners, honey, and syrups. Finally, subgroup analyses by age group, gender, race or ethnicity, education, family income, and BMI category separated for children or adolescents and adults were conducted to evaluate whether temporal trends in ASE differed by subgroup. All analyses were weighted to account for NHANES survey design and non-response bias. Analyses were done in Stata 16.0 (College Station, TX).

## Results

Characteristics of the sample are shown in Table 1 and are in-line with the US general population characteristics from 2001–2018. Mean total ASEs, total sugar, and the proportion of ASE from LCS-sweetened foods and beverages are also shown. Consumption of total ASE was highest among the 30–39-y age group while consumption of total sugars was highest among the 10–19-y age group. Overall, about 10.5% of ASE was estimated to come from LCS sources including foods, beverages, and tabletop sweeteners. The proportion of ASE from LCS sources increased with age up until the oldest age group, where it declined. Men had higher total ASE and total sugar values than women, but women had a higher proportion of their ASE from LCS sources. By race or ethnicity, total ASE values were highest among the non-Hispanic white population, who also had a greater proportion of ASE coming from LCS sources. The proportion of ASE coming from LCS sources was <5% for the non-Hispanic black and Mexican-American population. For family income, individuals with higher family incomes had a greater proportion of their ASE coming from LCS sources, but total ASE values did not vary much between groups. Similar trends were observed for education. For adult BMI, total ASE intakes were comparable across groups, but the proportion of ASE from LCS increased with higher BMIs. Similar trends were observed for children by BMI, though the proportion of ASE from LCS was lower. Differences in energy-adjusted ASE by population subgroup were much more modest than crude values.

**Table 1:** Population characteristics and estimated total ASEs, total sugars, and proportion of ASE from low-calorie-sweetened sources, 2001–2018.

			**Mean (standard error)**	
	**N**	**Weighted**	**Total**	**Total ASE, g/d**	**Total**	**Proportion of**
	**N**	**%**	**ASE, g/d**	**per 2000 kcal**	**sugars, g/d**	**ASE from LCS, %**
Total population	74,461	100.0	133.8 (0.6)	131.4 (0.7)	119.7 (0.5)	10.5 (0.2)
Age, y						
2–9	13,176	10.9	120.4 (0.9)***	140.7 (0.7)***	117.7 (0.8)**	2.3 (0.2)***
10–19	16,786	14.2	141.0 (1.1)	133.2 (0.9)	135.9 (1)***	3.6 (0.2)***
20–29	7,757	14.4	140.3 (1.8)	123.5 (1.2)***	131.6 (1.7)***	6.2 (0.4)***
30–39	7,512	13.6	144.5 (1.6)	128.7 (1.3)	127.6 (1.5)***	11.7 (0.5)***
40–49 [ref]	7,462	14.0	143.1 (1.7)	132.5 (2.0)	122.5 (1.4)	14.4 (0.6)
50–59	6,777	13.8	135.4 (1.9)**	135.7 (3.4)	111.5 (1.4)***	17.7 (0.6)***
60–69	7,251	9.9	120.7 (1.5)***	128.7 (1.4)	99.6 (1.1)***	17.5 (0.6)***
≥ 70	7,740	9.2	109.4 (1.0)***	128.6 (0.9)	96.3 (0.9)***	11.9 (0.4)***
Gender						
Female [ref]	37,853	51.2	119.9 (0.7)	137.6 (1.0)	105.4 (0.5)	12.1 (0.3)
Male	36,608	48.8	148.3 (0.9)***	124.9 (0.6)***	134.7 (0.8)***	9.1 (0.2)***
Race or ethnicity						
Non-Hispanic white [ref]	28,662	65.2	139.1 (0.8)	135.9 (0.9)	121.2 (0.7)	12.9 (0.3)
Non-Hispanic black	17,602	12.1	129.8 (1.2)***	129.1 (0.9)***	123.6 (1.2)	4.7 (0.2)***
Mexican-American	15,176	9.9	124.4 (1.3)***	121.7 (0.9)***	118.5 (1.2)	4.8 (0.3)***
Other Hispanic	6,109	5.5	125.2 (1.6)***	125.2 (1.4)***	117.0 (1.4)**	6.6 (0.5)***
Other or mixed race	6,912	7.3	111.3 (2.1)***	113.0 (1.3)***	103.3 (1.7)***	7.3 (0.7)***
Family income to poverty ratio^a^						
<1.00 [lower income]	17,751	17.1	133.0 (1.4)	132.3 (1.1)***	125.8 (1.3)***	5.4 (0.3)***
1–1.99	18,524	21.5	131.7 (1)*	132.3 (0.9)***	121.4 (0.9)***	7.8 (0.3)***
2–3.99	17,668	28.5	136.6 (1.1)	134.8 (1.6)***	121.9 (1.1)***	10.8 (0.4)***
≥4.00 [higher income] [ref]	14,995	32.9	134.6 (1)	128.0 (0.9)	114.4 (0.9)	15.0 (0.4)
Education (age ≥ 25 y)^a^						
< HS	10,549	16.4	126.7 (1.5)	131.7 (2.4)**	114.6 (1.3)***	9.6 (0.4)***
HS	9,277	23.5	137.2 (1.6)***	135.2 (2.9)***	119.8 (1.3)***	12.7 (0.5)***
Some college	11,246	30.2	137.9 (1.2)***	132.5 (1)***	117.6 (1.1)***	14.7 (0.5)*
≥ College [ref]	9,383	29.9	130.2 (1.2)	123.6 (1.1)	108.7 (1)	16.5 (0.5)
BMI (kg/m^2^), adults^a^						
Underweight: <18.5	718	1.7	140.4 (6.1)	126.7 (3.8)	134.5 (6.1)	4.2 (0.8)***
Healthy weight: 18–24.9 [ref]	12,190	29.4	131.3 (1.3)	125.8 (1.5)	120.2 (1.3)	8.4 (0.4)
Overweight: 25–29.9	14,756	33.1	134.2 (1.1)*	126.9 (1.0)	117.3 (0.9)*	12.6 (0.4)***
Obese: ≥ 30	16,131	35.9	137.1 (1.1)***	135.4 (1.7)***	113.3 (0.9)***	17.4 (0.4)***
BMI (kg/m^2^), children^a^						
Underweight (<5th percentile)	1,871	6.5	124.1 (2.2)***	134.2 (1.7)	121.9 (2.3)***	1.7 (0.3)*
Healthy weight (5–84.9 percentile[ref]	17,663	61.4	133.7 (1.0)	137.2 (0.8)	130.3 (0.9)	2.6 (0.2)
Overweight (85–94.9 percentile)	5,613	18.7	130.9 (1.7)	135.3 (1.4)	125.9 (1.6) **	3.8 (0.3)***
Obesity (≥95th percentile)	4,283	13.4	131.4 (1.9)	135.8 (1.3)	124.7 (1.8)**	5.1 (0.5)***

a *Numbers may not add up to the totals due to missing values*.

Asterisks refer to pairwise comparison to specified reference group [ref] as follows: ^***^p < 0.001; ^**^0.001 < p < 0.01; ^*^0.01 < p < 0.05.

Trends in ASE from LCS foods and beverages, total sugars, and total ASE overall and for beverages, foods, and tabletop sweeteners separately by survey cycle are shown in Table 2. Overall, a 23.2% decline (*p*-trend < 0.001 in all cases unless otherwise noted) in total ASE was observed from 2001–2002 to 2017–2018, whereas total sugars declined by 22.3% and ASE from LCS sources declined by 33%. Trends, but not absolute values, were directionally similar when disaggregating data by age. Compared to adults, proportionate declines in total ASE were stronger in children or adolescents (−26.6 vs. 22.1%). Overall trends in ASE were not meaningfully altered after adjusting for energy (−21.0%), age group (−22.3%), race or ethnicity (−22.0%), and all three simultaneously (−19.8%), indicating that changes in energy intakes and demographics over the study period do not explain the observed trends in overall ASE. Supplementary Table 2 provides trend data broken out by beverages, foods, and tabletop sweeteners. Total ASE from beverages declined by 36.7%, whereas tabletop sweeteners declined by 23.8%, while foods were generally stable. Declines in total ASE from beverages were somewhat stronger for children (−41.6%) than adults (−35.2%). No change was observed for the contribution of foods sweetened with LCS, but the absolute contribution was very low. For trends in total sugars, the decline was also driven by beverages (−37%) and tabletop sweeteners (−25%).

**Table 2:** Trends in total ASEs, total sugars, and ASE from LCS sources, overall and for children or adolescents and adults separately, 2001–2018.

	**Weighted mean g/d (standard error)**											
	**2001–2002**	**2003–2004**	**2005–2006**	**2007–2008**	**2009–2010**	**2011–2012**	**2013–2014**	**2015–2016**	**2017–2018**	***p*-trend**	**% change 2017–2018 vs. 2001–2002**	**Average annual change (SE)**
**Total population: age** **≥** **2 y**											
Total ASE, g/d	152 (1.6)	147 (2)	142 (2.7)	136 (2.4)	135 (1.4)	135 (1.5)	126 (1.8)	118 (1.6)	117 (1.6)	<0.001	−23.2%	−2.2 (0.11)
Total sugars, g/d	139 (1.7)	133 (1.6)	124 (2.3)	120 (1.9)	119 (1.1)	120 (1.3)	112 (1.3)	106 (1.5)	108 (1.4)	<0.001	−22.3%	−1.9 (0.10)
ASE from LCS sources, g/d	14 (1.2)	14 (1.3)	17 (1.1)	16 (0.7)	16 (0.8)	15 (0.9)	14 (0.8)	11 (0.9)	9.2 (0.8)	<0.001	−32.9%	−0.31 (0.07)
**Children and adolescents: (age 2–19 y)**											
Total ASE, g/d	154 (2.3)	152 (2.4)	143 (3.1)	133 (1.9)	133 (1.9)	133 (2.1)	119 (2.1)	111 (1.9)	113 (1.4)	<0.001	−26.6%	−2.8 (0.14)
Total sugars, g/d	151 (2.2)	148 (2.2)	138 (2.9)	128 (1.7)	127 (1.6)	128 (1.8)	114 (1.7)	108 (2.1)	110 (1.3)	<0.001	−26.6%	−2.7 (0.13)
ASE from LCS sources, g/d	3.1 (0.3)	4.0 (0.5)	4.5 (0.8)	5.3 (0.7)	5.6 (0.7)	4.6 (0.5)	4.7 (0.8)	2.3 (0.4)	2.3 (0.4)	0.02	−25.0%	−0.07 (0.03)
**Adults (age** **≥** **20 y)**											
Total ASE, g/d	152 (2.2)	145 (2.5)	141 (3)	137 (3.1)	136 (1.7)	135 (1.5)	129 (1.9)	120 (1.9)	118 (2.1)	<0.001	−22.1%	−2.00 (0.14)
Total sugars, g/d	134 (2.6)	127 (1.8)	120 (2.6)	117 (2.5)	117 (1.3)	117 (1.4)	111 (1.4)	106 (1.6)	107 (1.7)	<0.001	−20.4%	−1.58 (0.13)
ASE from LCS sources, g/d	18 (1.5)	18 (1.6)	22 (1.4)	20 (0.9)	19 (1.1)	18 (1.2)	17 (0.9)	14 (1.1)	11 (0.9)	<0.001	−35.4%	−0.42 (0.08)

The results of sensitivity analyses are shown in Supplemental Tables 3, 4. All sensitivity analyses were consistent with the observed significant decrease in the ASE observed in the primary analysis. The estimate informed by the sensory trial showed a change in the estimated annual change in estimated sweetness compared with the primary analysis of less than 1%. The only sensitivity analysis that deviated from the primary analysis by ± 2% (estimated annual change in ASE) was the analysis incorporating a 10-fold increase (from 1% to 20%) of foods within given categories that contained LCS, but even for this analysis the qualitative interpretation of a downward trend in ASE was unchanged. Additional sensitivity analyses excluding potential under-reporters of energy observed qualitatively similar trends in total ASE, total sugar, and ASE from LCS foods or beverages; the respective percent change in the primary and sensitivity analysis for total ASE was −23.2 and −21.2%, respectively (*p*-trend < 0.001 for both) (Supplementary Table 4).

Supplementary Table 5 provides estimates in the trends of ASE by population subgroup. Among adults, total ASE declined more dramatically among younger ages, with the declines among those aged ≥60 y not being statistically significant. Declines in ASE from LCS sources were observed for those aged 30–69 y with the biggest declines among those aged 30–49 y. Total ASE declines were larger for men as compared to women but declines in ASE from LCS sources were stronger for women. No heterogeneity was observed by race or ethnicity, with all groups experiencing a decline in total ASE. For both education and income, higher levels appeared to be associated with larger declines in total ASE. Among children or adolescents, those aged 10–19 y had greater declines than those aged 2–9 y as did men as compared to women. No differences in trends were observed by race or ethnicity, or family income. No major difference was observed for BMI, except for a decline in ASE from LCS among children or adolescents who were obese, but not for other groups.

**Figure 1:**
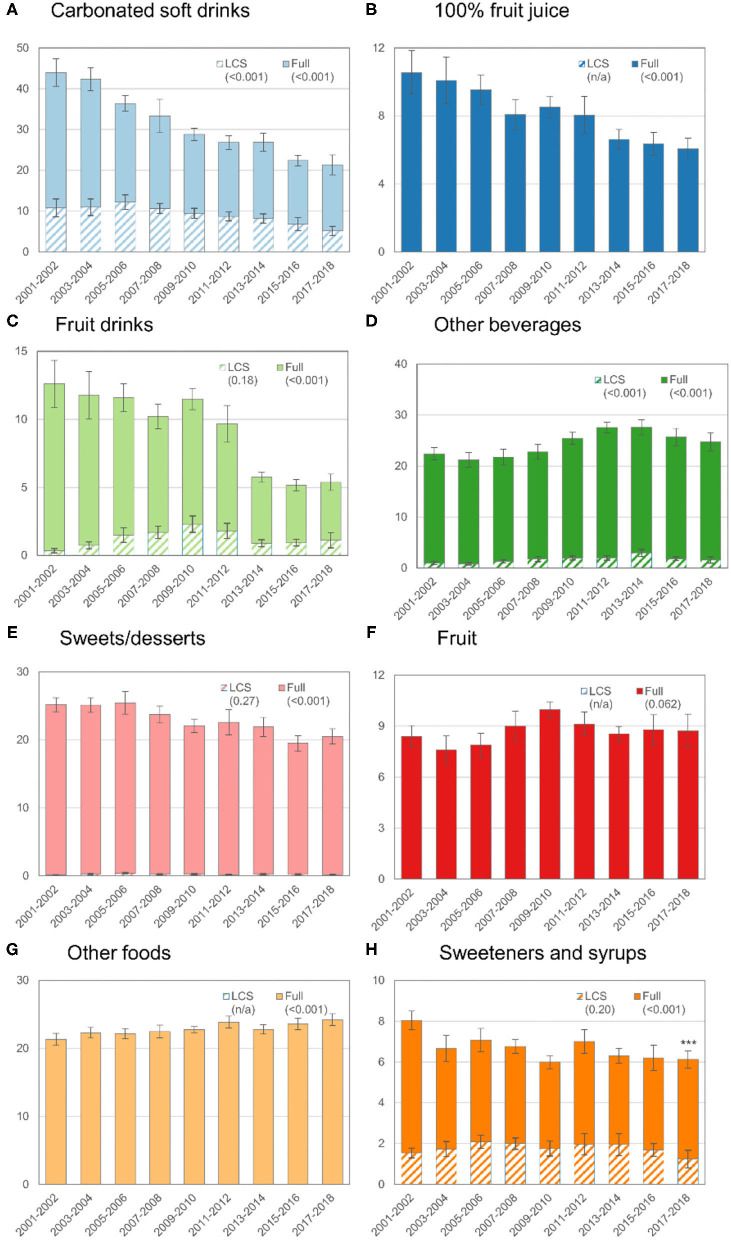
Trends in mean ASEs by food or beverage category in the total population (age ≥ 2 y), 2001–2018. The y-axis for each graph is the ASE value and the hashed bars indicate the ASE from LCS sources (e.g., diet soft drinks, dietetic cookies, or tabletop sweeteners). The solid bars correspond to the total sugar from that source (e.g., full). The error bars correspond to the 95% confidence interval for the corresponding bar. The values in parentheses are the *p*-value for trend. The *p*-value for the trend was not estimated when the contribution of LCS sources to the ASE was 0 or very low (e.g., for fruit, 100% fruit juice, and other foods).

**Figure 2:**
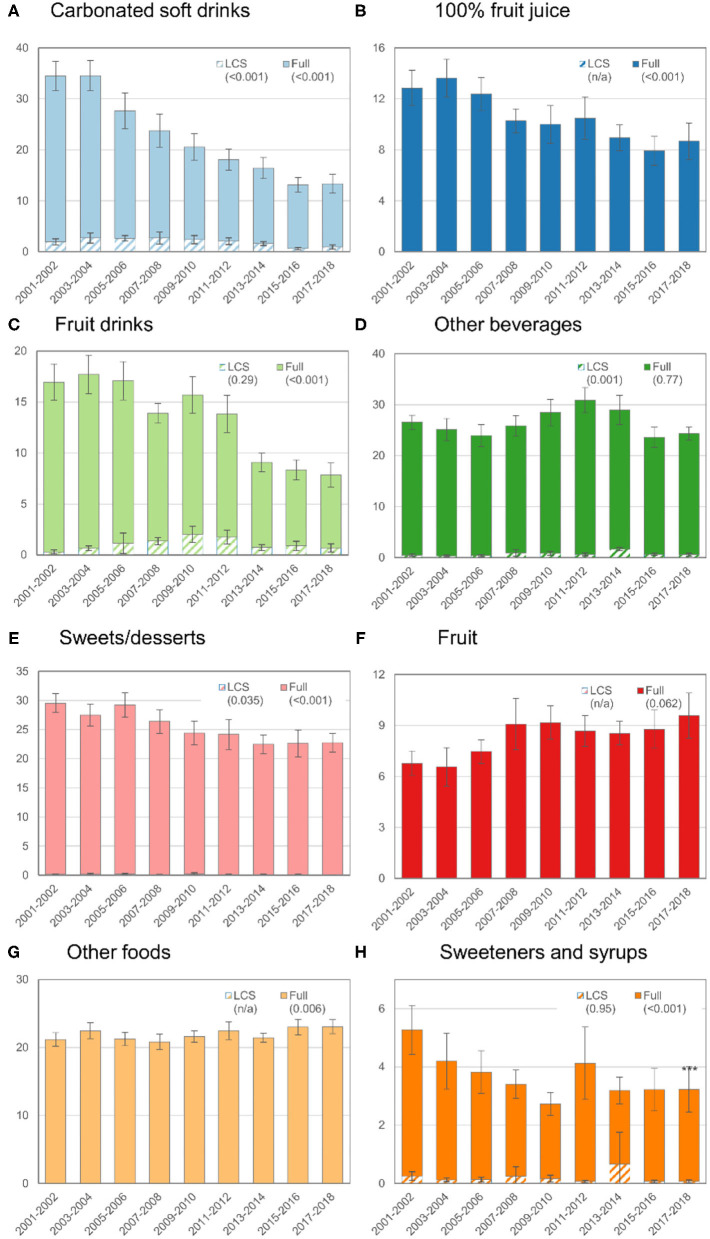
Trends in mean ASEs by food or beverage category among children or adolescents (age 2–19 y), 2001–2018. See footnote from Figure 1 for how to interpret this figure.

**Figure 3:**
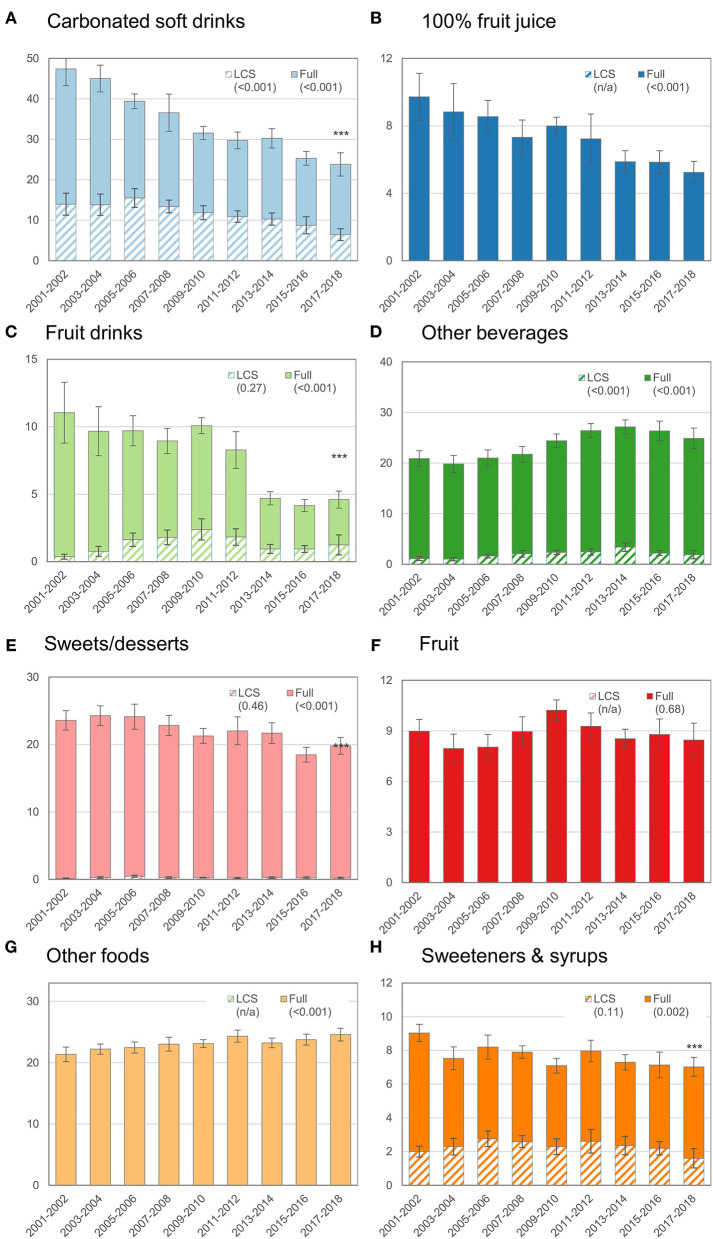
Trends in mean ASEs by food or beverage category among adults (age ≥ 20 y), 2001–2018. See footnote from Figure 1 for how to interpret this figure.

Figures 1–3 further disaggregate trends in ASE by food or beverage category for the overall population and for children (Figure 2) and adults (Figure 3) separately. Trends are separated for LCS vs. traditional sweeteners to identify potential diverging trends for the source of ASE. Figure 1A shows trends in ASE for CSDs and reveals a steady decline for both sugar and LCS-sweetened CSDs, though the proportional decline in sugar-sweetened CSDs is more marked. For some categories, such as fruit juice, sweets or desserts, whole fruit, and other foods, the contribution of LCS products was minimal or nonexistent. Overall, ASE from fruit juice and fruit drinks declined whereas ASE from other beverages increased through 2011–2014 and subsequently declined. Total ASE from the sweets or desserts category declined from 25.2 g/d to 20.5 g/d (*p*-trend < 0.001), but ASE from the other foods category increased slightly from 21.3 g/d to 24.2 g/d (*p*-trend < 0.001). For tabletop sweeteners and syrups, no trend was observed for LCS-sweetened products but a downward trend was observed for the overall category.

## Discussion

In this large nationally representative survey of more than 74,000 participants with detailed dietary data collected from 2001 to 2018, we observed a marked 23% decline in the total ASEs of the diet with significant declines for both total sugars and ASE from LCS sources. This decrease was observed among both adults and children and appears to be driven predominately by beverages, including CSDs, 100% fruit juice and fruit drinks. Modest declines in total ASE from tabletop sweeteners were also shown, though no meaningful changes in the overall contribution of foods to the total ASE of the diet were observed.

As many have noted, measuring dietary sweetness is notoriously challenging due in part to numerous factors affecting ratings of perceived sweetness intensity. Some of these factors include the impact of sweetener concentration, range or context of presented stimuli, food matrix, intraindividual variability of sweetness perception, and also one concept of sweetness (6). Our intent was to develop a pragmatic approximation that could be used to assess dietary trends in large population-based surveys and is not intended to replace other methodologies that may be better suited for the purpose (e.g., small-scale clinical studies or sensory trials). In addition, given that the level of LCS is not required to be displayed on nutrition labels in the US or included as a formal field in most dietary databases, including those used in NHANES, LCS consumption data were obtained relying on non-ingredient-specific product keywords. This likely resulted in some systematic mis-classification of LCS products, especially foods, which were much less likely to include relevant keywords. If a participant did consume an item sweetened with LCS but did not report this during the dietary recall the estimate of the ASE would not be affected if we assume approximate equivalence (e.g., yogurt sweetened with LCS being approximately equivalent to yogurt sweetened with caloric sweeteners), though the proportion of the ASE from LCS would be downwardly biased. Most importantly, the approach used to estimate the ASE has not been formally validated, and future investigations should aim to do so. Despite these important limitations, data from the sensory trial showed that the assumption of comparable perceived sweetness of the matched pairs was generally reasonable, especially for the most commonly consumed LCS items (e.g., regular cola vs. diet cola). Finally, multiple sensitivity analyses revealed that the observed major declines in population-level ASE were robust to random or systematic errors in the application of the matched pairs approach, exclusion of under-reporters and imposition of temporal misclassification of LCS in foods.

Our data contribute, and in some places stand in contrast, to a growing literature evaluating changes in the purchase and use of LCS and other sweeteners in the food supply. Sales data have shown that proportions of the US households purchasing products containing either LCS alone or in combination with caloric sweeteners have increased significantly from 65.7 to 67.2% and from 46.7 to 74.1%, respectively, between 2001 and 2018 (20). This increase in purchases of products containing LCS has occurred in tandem with a decline in purchases of products containing caloric sweeteners. The increase in purchases is suggested to be due to an increase in the number of products containing LCS entering the marketplace. The current finding of a 33% decline in ASE from LCS sources in the context of increased incorporation into several food and beverage items necessitates more research. One explanation for these findings is the difficulties in evaluating human consumption due to challenges extracting and the accuracy of the data from nutrient databases. For example, the USDA Food and Nutrient Database for Dietary Studies (FNDDS) list many food and beverage items as either containing a “LCS,” “dietetic,” or “sugar-free,” yet information about the specific LCS is not readily accessible. In addition, LCS are also included in other food and beverage items that do not clearly indicate the presence of LCS (11). An alternative explanation is that the number of products containing LCS may not be an accurate measure of population-level exposure due to a small number of products driving exposure (e.g., tabletop sweeteners and low-calorie beverages).

The decline in total ASE from beverages outpaced changes from foods and tabletop sweeteners. Much of this change is due to a decline in CSDs coming from both caloric sweeteners and LCS. The proportional decline in sugar-sweetened CSD was more marked than that for LCS CSDs, but apparent for both. Our study provides updated data on trends in CSDs, building upon prior work showing large declines in sugar-sweetened beverage (SSB) consumption, and particularly CSDs, among all ages from the late 1990s through the early 2010s (17, 21). Decreases in SSB consumption are driven by a combination of consumers drinking less SSBs and fewer consumers of SSBs overall (16). Efforts in both public health and clinical medicine have been made to reduce SSB consumption. National guidelines and initiatives have educated the public on the benefits of a healthy diet, including reducing SSB consumption. For example, the Dietary Guidelines for Americans have provided suggestions on cutting down on added sugars by replacing foods and drinks high in added sugars with healthier options such as drinking water or low-fat milk with meals instead of SSBs or just choosing smaller portions of SSBs (2). Some professional organizations, including the American Academy of Pediatrics and the American Heart Association, have also endorsed efforts to reduce the consumption of SSBs (22).

Additional trends in beverages in this study show that total ASE from fruit juice and fruit drinks also declined whereas ASE from other beverages increased between 2011 and 2014, but subsequently declined. This is consistent with previous population-based studies showing that among both youth and adults, fruit drink consumption has declined whereas consumption of sports and energy drinks and non-traditional SSBs such as sweetened coffees, teas, and blended dairy-based beverages has increased (21, 23). Growing popularity of non-traditional SSB may be due in part to perceptions that these beverages are healthier alternatives to traditional SSBs.

Compared to adults, proportionate declines in total ASE were somewhat stronger in children or adolescents (−26.6 vs. 22.1%) due primarily to more dramatic changes for ASE from beverages. Previous analyses of NHANES data have shown that there has been a sharp drop in added sugars intake and in SSB consumption by adolescents and young adults (16, 17, 24). There have been increases, since the early 2000s, in the number of federal, state, and local policies and campaigns aimed at reducing SSB intake, particularly in venues serving children and adolescents, such as schools and early childhood education centers. Another example is healthy kids' meals policies, which have been passed by many US states and cities that require restaurants to only offer healthier drinks (i.e., 100% juice, milk, or water).

For tabletop sweeteners and syrups, no trend was observed for LCS-sweetened products, but a downward trend was observed for the overall category. One explanation for these findings may be the increase in consumption of presweetened foods and beverages away from home. According to Drewnowski et al., store-bought added sugars accounted for 65 to 75% of added sugars consumed, depending on age whereas the total amount of added sugars from restaurants, school cafeterias, and other sources was considerably lower (25).

Despite trends in beverages and tabletop sweeteners and syrups, the overall contribution of foods to total ASE did not meaningfully change, though it did decline for sweets or desserts while increasing slightly for other foods. Important sources of ASE for the other foods category include mixed dishes, yeast breads, ready-to-eat cereals, tomato sauces, sandwiches, quick breads, and salad dressings. This is consistent with previous population studies showing that beverages continue to comprise the majority of reported added sugars or LCS consumption (11, 26, 27). According to the Dietary Guidelines for Americans, the top source of added sugars in the US comes from SSBs at 24% (2). For food sources of added sugars, desserts and sweet snacks comprise 19% followed by candy (9%), breakfast cereals and bars (7%), and yogurt (4%). Recent sales data have shown notable but relatively small increases in the amount of the top 10 food groups in the US household with products containing LCS compared with what was seen for beverages, between 2001 and 2018 (20), suggesting growth of LCS-containing food use over time, albeit from a small baseline. While we did not observe an increase in ASE from LCS food sources, we do show that per our methodology, almost all sweetness from foods comes from naturally occurring sugar or added caloric sweeteners and not from LCS sources.

Beyond trends, the present data further examined ASE of the diet overall and contribution from total sugars or LCS by sociodemographic characteristics. Here, we estimate that overall, about 10.5% of ASE was from LCS sources. This finding adds to previous reports showing that about 11.3% of all eating episodes contained some items that contained LCS (28). Further, consistent with prior population-based studies, total sugar consumption was highest among children and adolescents which declined with age as the proportion of ASE from LCS sources simultaneously increased until the oldest age group, where it declined slightly (11, 16, 26, 28). Consistent with prior literature that did not attempt to enumerate LCS amounts, the proportional contribution of sweetness from LCS sources was highest among women, the non-Hispanic white population, the more highly educated, individuals with higher family incomes, and those with higher BMIs. The consistency of these patterns across population subgroups provides some indirect support of our methodology. In addition, consistent with prior literature, the mean total sugar intake was highest among men, the non-Hispanic black population, the less educated and individuals with lower socioeconomic status (16, 26). Despite the differences in contribution of source (caloric or LCS) to ASE by sociodemographic characteristics, overall ASE values did not vary dramatically between groups. This was observed for family income, education, and also BMI, where marked trends in the contribution of total sugars or LCS were observed, but not for total sweetness, suggesting that studies assessing only a single source of sweetness (e.g., added sugars, total sugars, or LCS sources) may not accurately identify population subgroup differences in terms of dietary sweetness exposure.

Despite observed declines in total dietary sweetness, the bigger questions regarding the nutrition and health implications of sweet taste still need to be addressed. Health policy recommendations on reducing all sweetness in the diet, whether that be from naturally occurring sugar, added sugars or LCS, stem from unconfirmed hypotheses that consumption of sweet things train palates to crave sweetness leading to obesity and metabolic dysfunction (6). Whereas our study was not designed to formally establish the role of dietary sweetness on risk of obesity, the increasing prevalence of obesity observed over the past two decades among both children (15.4 to 19.3% from 2001to 2018) and adults (30.5 to 42.4% from 2001 to 2018) stands in contrast to the observed declines in dietary sweetness (−23% change) (29, 30). This is the first study to estimate sweetness of the diet including sweet taste coming from LCS, moving beyond the use of taste database methodology. It is also the first study to formally enumerate trends and subpopulation differences in potential exposure to LCS in terms of sweetness rather than simply looking at the proportion of individuals consuming a given type of food or beverage. This practical approach and underlying data may help researchers to apply the technique to other dietary studies to further explore the nutrition and health implications of dietary sweetness.

Beyond the previously noted limitations of the sweetness algorithm, additional limitations should be noted. First, this study was based on a single 24-h dietary recall, which does not allow us to characterize the population distribution of ASE and only allows us to evaluate mean intakes. Second, dietary recalls such as all self-report dietary assessment tools are subject to potential underreporting, which could bias study results (19). Furthermore, individuals tend to underreport the consumption of foods and beverages perceived to be less healthful by underestimating amounts eaten or omitting them altogether, which may result in a falsely minimized estimation of the ASE (31). Sensitivity analyses excluding possible under-reporters of dietary energy observed similar proportional declines in estimated dietary sweetness over the study period. While self-reported dietary data will always be subjected to both random and systematic errors, our approach would only be feasible when rich and detailed food data are collected, such as in a 24-h recall, and would not be possible with more crude instruments such as a food frequency questionnaire. Strengths of the study include the large sample size and population-based design, which allowed us to generalize to the US child and adult populations while also conducting detailed subgroup assessments.

## Conclusions

In this large population-based study, we observed a marked decrease in the total ASE of the diet with significant declines for both total sugars and ASE from LCS sources. Declines in total ASE were predominantly driven by beverages, namely CSDs, 100% fruit juice and fruit drinks. Reductions in tabletop sweeteners were also observed but no meaningful changes overall for ASE from foods. This was the first study to estimate sweetness of the diet including sweet taste coming from LCS, moving beyond the use of taste database methodology. While this serial cross-sectional study is not designed to establish the role of dietary sweetness on obesity risk, the declining dietary sweetness and increasing obesity rates suggest that this is an area deserving greater research attention. The practical approach used here, and underlying data may help researchers to apply the technique to other dietary studies to further address these questions.

## Data Availability Statement

Publicly available datasets were analyzed in this study. This data can be found here: https://wwwn.cdc.gov/nchs/nhanes/Default.aspx.

## Author Contributions

AK and CDR: methodology, conceptualization, formal analysis, writing-original draft, writing, reviewing, and editing. ARW: methodology, conceptualization, formal analysis, writing, reviewing, and editing. All authors contributed to the article and approved the submitted version.

## Funding

This study was funded by PepsiCo, Inc. The funder was not involved in study design, methodology development, analysis, interpretation of data, or the writing of this article but did review the article approving it for publication.

## Author Disclaimer

The views expressed in this manuscript are of those authors and do not necessarily reflect the position or policy of PepsiCo, Inc.

## Author Disclaimer

The views expressed in this manuscript are of those authors and do not necessarily reflect the position or policy of PepsiCo, Inc.

## Conflict of Interest

AK, ARW, and CDR are salaried employees of PepsiCo, Inc.

## Publisher's Note

All claims expressed in this article are solely those of the authors and do not necessarily represent those of their affiliated organizations, or those of the publisher, the editors and the reviewers. Any product that may be evaluated in this article, or claim that may be made by its manufacturer, is not guaranteed or endorsed by the publisher.
