# Corrigendum: Estimated Sweetness in US Diet Among Children and Adults Declined From 2001 to 2018: A Serial Cross-Sectional Surveillance Study Using NHANES 2001–2018

**DOI:** 10.3389/fnut.2022.877571

**Published:** 2022-03-14

**Authors:** Alison Kamil, Alissa R. Wilson, Colin D. Rehm

**Affiliations:** ^1^Health & Nutrition Sciences, Life Sciences, PepsiCo R&D, Chicago, IL, United States; ^2^Health & Nutrition Sciences, Life Sciences, PepsiCo R&D, Purchase, NY, United States

**Keywords:** sugar-sweetened beverages, artificially sweetened beverages, nutrition surveys, cross-sectional studies, trends, United States, National Health and Nutrition Examination Survey, non-nutritive sweeteners

In the original article, there was a mistake in Figures 1, 2. The figures were uploaded in an incorrect order which resulted in a discrepancy between the figure captions and the figure images. Corrected versions of Figures 1, 2 appear below.

**Figure 1:**
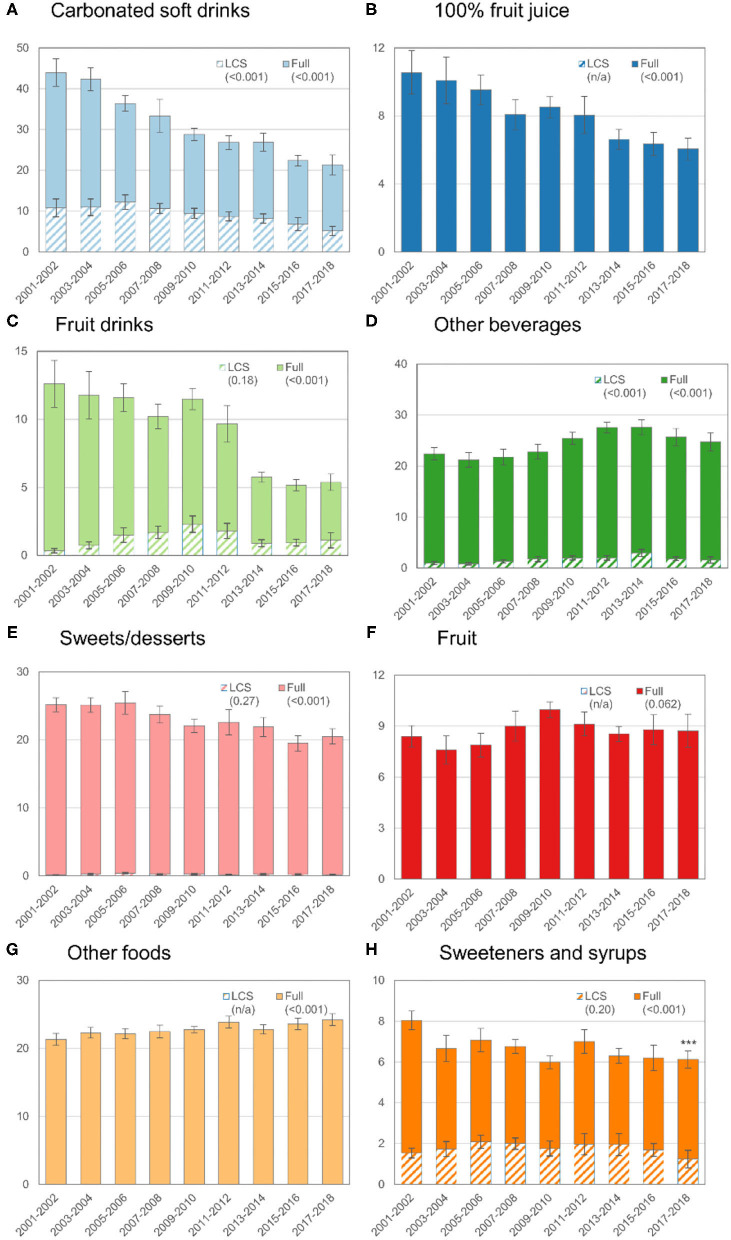
Trends in mean ASEs by food or beverage category in the total population (age ≥ 2 y), 2001–2018. The y-axis for each graph is the ASE value and the hashed bars indicate the ASE from LCS sources (e.g., diet soft drinks, dietetic cookies, or tabletop sweeteners). The solid bars correspond to the total sugar from that source (e.g., full). The error bars correspond to the 95% confidence interval for the corresponding bar. The values in parentheses are the *p*-value for trend. The *p*-value for the trend was not estimated when the contribution of LCS sources to the ASE was 0 or very low (e.g., for fruit, 100% fruit juice, and other foods).

**Figure 2:**
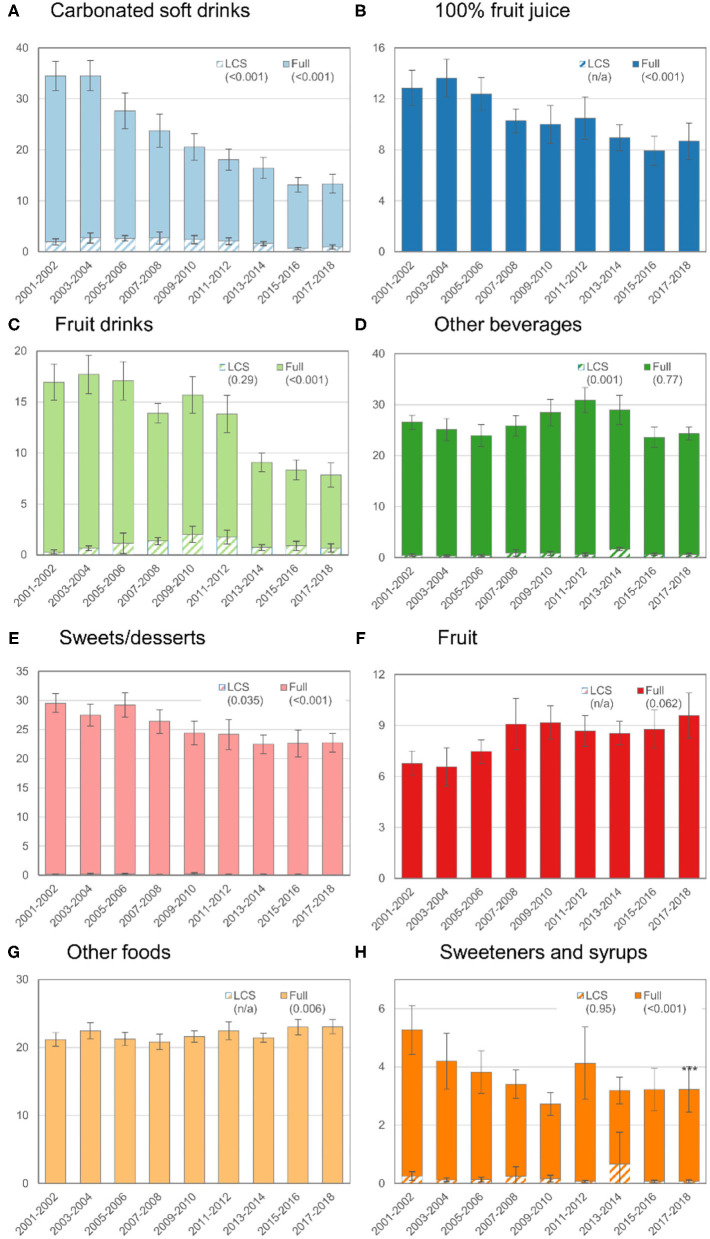
Trends in mean ASEs by food or beverage category among children or adolescents (age 2–19 y), 2001–2018. See footnote from Figure 1 for how to interpret this figure.

The authors apologize for this error and state that this does not change the scientific conclusions of the article in any way. The original article has been updated.

## Publisher's Note

All claims expressed in this article are solely those of the authors and do not necessarily represent those of their affiliated organizations, or those of the publisher, the editors and the reviewers. Any product that may be evaluated in this article, or claim that may be made by its manufacturer, is not guaranteed or endorsed by the publisher.

