# Supplementary material for: Estimated Sweetness in US Diet Among Children and Adults Declined From 2001 to 2018: A Serial Cross-Sectional Surveillance Study Using NHANES 2001–2018
Source: Front Nutr. 2021 Dec 17;8:777857. doi: 10.3389/fnut.2021.777857 (PMC8718635; doi:10.3389/fnut.2021.777857)
Supplement: Supplementary file 1 [file Data_Sheet_1.docx]

Supplementary Material

**Estimated sweetness in US diet amongst children and adults declined from 2001-2018: A serial cross-sectional surveillance study using NHANES 2001-2018**

**Contents**

[**Sensory Trial Methods** 2](#_Toc87002046)

[**Sensory Trial Results** 2](#_Toc87002047)

[**Supplemental Table 2.** Trends in approximate sugar equivalents (ASE) separating beverages, foods and tabletop sweeteners overall and for children/adolescents and adults separately, 2001-2018 5](#_Toc87002048)

[**Supplemental Table 3.** Results of sensitivity analyses on trends in approximate sugar equivalents (ASE), g/d 8](#_Toc87002049)

[**Supplemental Table 4.** Sensitivity analyses excluding potential under-reporters^a^ of total energy 10](#_Toc87002050)

[**Supplemental Table 5.** Trends in approximate sugar equivalents (ASE) by population sub-group 11](#_Toc87002051)

[**References** 14](#_Toc87002052)

**Sensory Trial Methods**

*Samples*

The list of sweet tasting product categories were chosen based on those contributing >~1% of overall LCS consumption (in terms of frequency) from NHANES 2015-2016, capturing >85% of the product categories in question. The specific matched sweet tasting products (full sugar vs. LCS equivalent) were identified across different large national brands. The list of products is shown in **Supplemental Table 1**. The products were purchased by Sensory Spectrum, Inc. (New Providence, NJ) and any products that needed prep were prepared according to manufacturer’s instructions. All beverages were served cold and 4 oz. per sample was served to each panelist.

*Procedure*

The samples were analyzed for sweetness intensity by 8-10 members of the Sensory Spectrum Food Panel, trained and experienced in flavor evaluation. The strength of each attribute was rated on the 15-point Spectrum Scale, where 0 = none and 15 = very strong.​This scale incorporates the ability to use tenths of a point and therefore has the potential of 150 scale differentiations (Hootman 1992). The panelists evaluated each sample in a randomized order and recorded individual ratings for each attribute in RedJade® software (RedJade Sensory Solutions, LLC, Martinez, CA). All samples were expectorated. Panelists cleared with water in between each sample as well as unsalted crackers, if needed. The next sample was brought out when all panelists had had enough time to clear.

*Data Analysis*

Univariate Analysis of Variance (ANOVA) was used to analyze differences in in-mouth sweet taste (highest point) between matched samples. The model included sample (fixed effect) and panelist (random effect). Significant differences among the samples are reported at both the 95% and the 90% confidence levels. ​Sample means were then compared using Fisher’s Least Significant Differences (95% CL and 90% CL) to identify which samples differ from each other. XLStat Statistical Software for Excel was used for all statistical tests. The significance level was set at α < 0.05 and < 0.10 for 95% and 90% CL respectively for the ANOVA calculations.

**Sensory Trial Results**

Differences in average in-mouth sweet taste (highest point) between matched (full sugar vs. LCS equivalent) sweet tasting products are shown in **Supplemental Table 1**. No significant differences were observed for sweeteners. For beverages, no significant differences were observed for carbonated soft drinks and energy drinks. Significant differences were observed for 1 set of fruit drink (p<0.001), 1 set of iced tea (p=0.004) as well as the sport drink (p=0.018) matched products. And for foods, significant differences were observed for 4 out of the 5 categories (creamer p=0.001, gelatin dessert p=0.004, jam p=0.004, and wafer cookie p=0.004). No significant differences were observed for yogurt. Across all 21 matched products, 2 items were rated as being equivalent, 10 were rated as the full sugar product being sweeter and 9 were rated as the LCS product being sweeter. This suggests, that while individual foods may vary, that on balance, differences in sweetness are generally comparable across different products and categories. **Supplemental Table 1.** Differences in average in-mouth sweet taste (highest point) between matched (full sugar vs. LCS equivalent) sweet tasting products

| **Alias name** | **Brand name** | **Full sugar** | **LCS equivalent** | **p-value of difference** |
| --- | --- | --- | --- | --- |
| **Sweeteners** | Average | 4.5 | 4.4 | n/a |
| Aspartame | Domino Sugar vs. Equal | 4.5 | 4.0 | 0.465 |
| Sucralose | Domino Sugar vs. Splenda | 4.5 | 4.5 |  |
| Saccharin | Domino Sugar vs. Sweet N Low | 4.5 | 5.1 |  |
| Stevia | Domino Sugar vs. Stevia in the Raw | 4.5 | 3.8 |  |
| **Beverages** | | | | |
| **Carbonated soft drinks** | Average | 9.2 | 9.2 | n/a |
| Cola | Pepsi vs. Diet Pepsi | 9.2 | 8.4 | 0.233 |
| Lemon-lime | Sprite vs. Sprite Zero Sugar | 9.1 | 9.2 | 0.813 |
| Ginger ale | Canada Dry vs. Canada Dry Zero Sugar | 7.1 | 9.4 | 0.093 |
| Pepper-style | Dr. Pepper vs. Diet Dr. Pepper | 10.1 | 9.6 | 0.214 |
| Citrus | Mountain Dew vs. Diet Mountain Dew | 10.3 | 9.6 | 0.282 |
| **Fruit drinks** | Average | 7.7 | 8.8 | n/a |
| Lemonade | Country Time Lemonade vs. Country Time Lemonade Zero | 6.2 | 8.6 | <0.001 |
| Fruit punch | Minute Maid Fruit Punch vs. Minute Maid Zero Sugar Fruit Punch | 9.1 | 8.9 | 0.718 |
| **Iced teas** | Average | 6.7 | 7.4 | n/a |
| Lemon iced tea 1 | Pure Leaf Lemon vs. Diet Pure Leaf Lemon | 6.4 | 8.0 | 0.004 |
| Lemon iced tea 2 | Snapple Lemon Tea vs. Diet Snapple Lemon Tea | 7.0 | 6.8 | 0.553 |
| **Energy drinks** | Average | 9.8 | 9.9 | n/a |
| Energy drink 1 | Red Bull vs. Red Bull Sugar Free | 8.5 | 8.5 | 0.925 |
| Energy drink 2 | Monster Energy vs. Monster Energy Zero Sugar | 11.0 | 11.3 | 0.763 |
| **Lemon Lime Sports drink** | Gatorade Lemon Lime vs. Gatorade G Zero Sugar Lemon Lime | 4.9 | 6.0 | 0.018 |
| **Foods** | | | | |
| Vanilla creamer | International Delight French Vanilla Creamer vs. International Delight French Vanilla Creamer Zero Sugar | 19.6 | 11.5 | 0.001 |
| Vanilla yogurt | Yoplait Original French Vanilla vs. Yoplait Light Very Vanilla | 8.3 | 9.6 | 0.052 |
| Strawberry gelatin dessert | Jell-O Strawberry Cup vs. Jell-O Strawberry Cup Sugar-Free | 6.9 | 8.9 | 0.004 |
| Strawberry jam | Smucker’s Strawberry Jam vs. Smucker’s Sugar-Free Strawberry Jam | 13.8 | 9.2 | 0.004 |
| Vanilla wafer cookie | Voortman’s Vanilla Wafer vs. Voortman’s Sugar-Free Vanilla Wafer | 14.0 | 10.5 | 0.004 |

**Supplemental Table 2.** Trends in approximate sugar equivalents (ASE) separating beverages, foods and tabletop sweeteners overall and for children/adolescents and adults separately, 2001-2018

|  | Weighted mean g/d (Standard Error) | | | | | | | | |  |  |  |
| --- | --- | --- | --- | --- | --- | --- | --- | --- | --- | --- | --- | --- |
|  | 2001-02 | 2003-04 | 2005-06 | 2007-08 | 2009-10 | 2011-12 | 2013-14 | 2015-16 | 2017-18 | p-trend | % change 2017-18 vs. 2001-02 | Average annual change (Std err) |
|  |  |  |  |  |  |  |  |  |  |  |  |  |
| **Total population: age≥2y** |  |  |  |  |  |  |  |  |  |  |  |  |
|  |  |  |  |  |  |  |  |  |  |  |  |  |
| Total ASE, g/d | | | | | | | | | | |  |  |
| Beverages | 89  (1.7) | 85  (2.1) | 78  (2.1) | 74  (2.4) | 73  (1.2) | 71  (1.2) | 66  (1.5) | 59  (1.5) | 56  (1.7) | <0.001 | -36.7% | -1.97  (0.11) |
| Foods | 56  (0.8) | 56  (1) | 56  (1.2) | 56  (1.1) | 56  (0.6) | 56  (1.2) | 54  (1.1) | 53  (1) | 55  (1.1) | 0.038 | -1.5% | -0.13  (0.06) |
| Tabletop sweeteners | 8.0  (0.3) | 6.7  (0.3) | 7.1  (0.4) | 6.8  (0.2) | 6.0  (0.3) | 7.0  (0.5) | 6.3  (0.4) | 6.2  (0.4) | 6.1  (0.3) | <0.001 | -23.8% | -0.08  (0.02) |
|  |  |  |  |  |  |  |  |  |  |  |  |  |
| Total sugars^a^, g/d |  |  |  |  |  |  |  |  |  |  |  |  |
| Beverages | 77  (1.5) | 72  (1.7) | 63  (1.8) | 59  (2) | 60  (1) | 59  (1.2) | 54  (1.4) | 49  (1.3) | 48  (1.5) | <0.001 | -37.0% | -1.7  (0.10) |
| Foods | 55  (0.8) | 55  (1) | 56  (1.2) | 56  (1.1) | 55  (0.6) | 56  (1.2) | 54  (1.1) | 53  (1) | 55  (1.1) | 0.042 | -1.6% | -0.13  (0.06) |
| Tabletop sweeteners | 6.5  (0.2) | 4.9  (0.3) | 5.0  (0.3) | 4.8  (0.2) | 4.2  (0.2) | 5.0  (0.3) | 4.4  (0.2) | 4.5  (0.3) | 4.9  (0.2) | <0.001 | -24.8% | -0.07  (0.02) |
|  |  |  |  |  |  |  |  |  |  |  |  |  |
| ASE from LCS sources, g/d | | | | | | | | | | | | |
| Beverages | 12  (1.2) | 13  (1.2) | 15  (1) | 14  (0.8) | 14  (0.8) | 12  (0.8) | 12  (0.6) | 9.5  (0.9) | 7.8  (0.7) | <0.001 | -35.3% | -0.29 (0.06) |
| Foods | 0.1  (0.1) | 0.2  (0.1) | 0.4  (0.1) | 0.2  (0.1) | 0.2  (0.1) | 0.2  (0.1) | 0.2  (0.1) | 0.2  (0.1) | 0.2  (0.1) | 0.47 | 34.9% | -0.002 (0.003) |
| Tabletop sweeteners | 1.5  (0.1) | 1.7  (0.2) | 2.1  (0.2) | 2.0  (0.1) | 1.8  (0.2) | 2.0  (0.3) | 1.9  (0.3) | 1.7  (0.2) | 1.2  (0.2) | 0.20 | -19.7% | -0.02 (0.012) |
|  |  |  |  |  |  |  |  |  |  |  |  |  |
|  |  |  |  |  |  |  |  |  |  |  |  |  |
| **Children & adolescents: age2-19y** |  |  |  |  |  |  |  |  |  |  |  |  |
|  |  |  |  |  |  |  |  |  |  |  |  |  |
| Total ASE, g/d | | | | | | | | | | |  |  |
| Beverages | 90  (2) | 90  (2) | 80  (3.1) | 73  (1.7) | 74  (1.4) | 72  (1.2) | 62  (1.7) | 52  (1.1) | 53  (1.4) | <0.001 | -41.6% | -2.5  (0.12) |
| Foods | 58  (1.1) | 57  (1.1) | 59  (1.0) | 57  (1.5) | 56  (1.0) | 56  (1.4) | 54  (1.1) | 55  (1.8) | 57  (1.0) | 0.026 | -2.1% | -0.18  (0.08) |
| Tabletop sweeteners | 5.3  (0.4) | 4.2  (0.5) | 3.8  (0.4) | 3.4  (0.3) | 2.7  (0.2) | 4.1  (0.6) | 3.2  (0.6) | 3.2  (0.4) | 3.2  (0.4) | 0.001 | -38.7% | -0.10  (0.03) |
|  |  |  |  |  |  |  |  |  |  |  |  |  |
| Total sugars^a^, g/d |  |  |  |  |  |  |  |  |  |  |  |  |
| Beverages | 88  (1.9) | 87  (1.8) | 76  (2.9) | 68  (1.6) | 69  (1.1) | 68  (1.1) | 58  (1.6) | 50  (1.3) | 51  (1.3) | <0.001 | -42.3% | -2.5  (0.11) |
| Foods | 58  (1.2) | 57  (1.1) | 58  (1.0) | 57  (1.5) | 56  (0.9) | 56  (1.4) | 53  (1.1) | 55  (1.8) | 57  (1.0) | 0.03 | -2.0% | -0.17  (0.08) |
| Tabletop sweeteners | 5.0  (0.4) | 4.1  (0.5) | 3.7  (0.4) | 3.2  (0.2) | 2.6  (0.2) | 4.1  (0.6) | 2.5  (0.2) | 3.2  (0.4) | 3.2  (0.4) | <0.001 | -36.9% | -0.10  (0.03) |
|  |  |  |  |  |  |  |  |  |  |  |  |  |
| ASE from LCS sources, g/d | | | | | | | | | |  |  |  |
| Beverages | 2.7  (0.3) | 3.7  (0.5) | 4.2  (0.8) | 5  (0.6) | 5.2  (0.7) | 4.4  (0.5) | 3.9  (0.3) | 2.1  (0.4) | 2.2  (0.4) | 0.015 | -19.4% | -0.07 (0.03) |
| Foods | 0.1  (0) | 0.1  (0.1) | 0.2  (0.1) | 0.1  (0.1) | 0.2  (0.1) | 0.1  (0.1) | 0.1  (0.1) | 0.1  (0.1) | 0.1  (0.1) | 0.17 | -43.6% | -0.003 (0.003) |
| Tabletop sweeteners | 0.3  (0.1) | 0.1  (0.1) | 0.1  (0.1) | 0.2  (0.2) | 0.2  (0.1) | 0.1  (0.1) | 0.7  (0.6) | 0.1  (0.1) | 0.1  (0.1) | 0.95 | -75.2% | -0.0006 (0.01) |
|  |  |  |  |  |  |  |  |  |  |  |  |  |
| **Adults**  **(age≥20y)** |  |  |  |  |  |  |  |  |  |  |  |  |
|  |  |  |  |  |  |  |  |  |  |  |  |  |
| Total ASE, g/d | | | | | | | | | | |  |  |
| Beverages | 88  (2.1) | 83  (2.7) | 78  (2.1) | 74  (3) | 73  (1.4) | 71  (1.4) | 67  (1.5) | 61  (1.9) | 57  (2) | <0.001 | -35.2% | -1.78  (0.14) |
| Foods | 55  (1.2) | 55  (1.1) | 56  (1.4) | 56  (1.1) | 56  (0.7) | 57  (1.2) | 54  (1.2) | 52  (0.9) | 54  (1.3) | 0.026 | -1.1% | -0.11  (0.08) |
| Tabletop sweeteners | 9.0  (0.4) | 7.5  (0.3) | 8.2  (0.5) | 7.9  (0.3) | 7.1  (0.4) | 8.0  (0.5) | 7.3  (0.4) | 7.1  (0.5) | 7.0  (0.4) | 0.001 | -22.3% | -0.09  (0.03) |
|  |  |  |  |  |  |  |  |  |  |  |  |  |
| Total sugars^a^, g/d |  |  |  |  |  |  |  |  |  |  |  |  |
| Beverages | 73  (2) | 67  (2.1) | 59  (1.9) | 56  (2.3) | 57  (1.1) | 56  (1.4) | 52  (1.4) | 49  (1.5) | 48  (1.8) | <0.001 | -34.5% | -1.39  (0.13) |
| Foods | 55  (1.2) | 55  (1.1) | 55  (1.4) | 55  (1) | 55  (0.7) | 56  (1.2) | 54  (1.2) | 52  (0.9) | 54  (1.3) | 0.14 | -1.2% | -0.11  (0.08) |
| Tabletop sweeteners | 7.0  (0.3) | 5.2  (0.3) | 5.4  (0.4) | 5.3  (0.2) | 4.8  (0.2) | 5.4  (0.3) | 4.9  (0.2) | 4.9  (0.4) | 5.4  (0.3) | 0.002 | -22.9% | -0.07  (0.02) |
|  |  |  |  |  |  |  |  |  |  |  |  |  |
| ASE from LCS sources, g/d | | | | | | | | | |  |  |  |
| Beverages | 15  (1.5) | 16  (1.5) | 19  (1.3) | 17  (1.0) | 17  (1.0) | 15  (1.0) | 15  (0.8) | 12  (1.2) | 9.6  (0.8) | <0.001 | -38.1% | -0.39  (0.08) |
| Foods | 0.1  (0) | 0.3  (0.1) | 0.4  (0.1) | 0.3  (0.1) | 0.2  (0.1) | 0.2  (0.1) | 0.3  (0.1) | 0.2  (0.1) | 0.2  (0.1) | 0.63 | 59.2% | -0.002  (0.004) |
| Tabletop sweeteners | 2.0  (0.2) | 2.3  (0.3) | 2.8  (0.2) | 2.6  (0.2) | 2.3  (0.2) | 2.6  (0.4) | 2.4  (0.3) | 2.2  (0.2) | 1.6  (0.3) | 0.11 | -20.0% | -0.024  (0.02) |
|  |  |  |  |  |  |  |  |  |  |  |  |  |

^a^ Exclusive of approximate sugar equivalents from LCS foods and beverages

**Supplemental Table 3.** Results of sensitivity analyses on trends in approximate sugar equivalents (ASE), g/d

|  | Annual change (SE) | % Change from primary analysis | p-trend |
| --- | --- | --- | --- |
| Primary analysis | -2.19 (0.12) | - | <0.001 |
| Estimate based on sensory trial data | -2.17 (0.11) | -0.81% | <0.001 |
| Random +/- 20% of LCS sweetened foods following uniform distribution | -2.19 (0.12) | +0.07% | <0.001 |
| Systematic under-estimate of 0-20% following uniform distribution | -2.21 (0.12) | -1.26% | <0.001 |
| Systematic over-estimate of 0-20% following uniform distribution | -2.16 (0.11) | +1.29% | <0.001 |
| Excluding under-reporters^a^ | -2.23 (0.13) | -1.7% | <0.001 |
| Systematic mis-classification of foods with LCS^b^ | -2.13 (0.12) | +2.7% | <0.001 |

^a^ Detailed results for this analysis are available in Supplemental Table 4, but are provided here for comparison to the other sensitivity analyses.

^b^ This sensitivity analysis addresses potential concerns that foods with LCS may be more difficult to identify in NHANES than beverages or tabletop sweeteners and that consumption of such foods may have increased over the study period. Briefly, we identified the common food categories containing LCS identified by Dunford et al (Dunford, Miles et al. 2020) and systematically imposed a linear trend in the proportion of foods that may have contained LCS from 1% in 2001-2002 to 10% in 2017-2018 and scaling this proportion linearly across this period. We then used a uniform distribution to increase the approximate sugar equivalents of these foods by between 10-20%. The food groups evaluated included yogurt, dairy-based desserts, grain-based desserts, candy, other dairy items (e.g., whipped cream and creamers), bars, bread and bread products, sauces and canned/jarred fruit. In NHANES these food groups were identified by their FNDDS prefix code (e.g., 51 for bread products), by manually querying the food descriptions (e.g., for canned fruit) or a combination of the two approaches (e.g., for bars).

**Supplemental Table 4.** Sensitivity analyses excluding potential under-reporters^a^ of total energy

|  | Weighted mean g/d (standard error) | | | | | | | | |  |  |  |
| --- | --- | --- | --- | --- | --- | --- | --- | --- | --- | --- | --- | --- |
|  | 2001-02 | 2003-04 | 2005-06 | 2007-08 | 2009-10 | 2011-12 | 2013-14 | 2015-16 | 2017-18 | p-trend | % change 2017-18 vs. 2001-02 | Average annual change (SE) |
|  |  |  |  |  |  |  |  |  |  |  |  |  |
| **Total population: age≥2y** |  |  |  |  |  |  |  |  |  |  |  |  |
|  |  |  |  |  |  |  |  |  |  |  |  |  |
| Total approximate sugar equivalents (ASE), g/d | 170 (1.5) | 163 (2.5) | 156 (2.5) | 154 (3.1) | 149 (1.5) | 149 (1.4) | 142 (1.6) | 133 (2.1) | 134 (1.9) | <0.001 | -21.2% | -2.2  (0.13) |
| Total sugars, g/d | 157 (1.8) | 149 (2.0) | 140 (2.4) | 139 (2.7) | 135 (1.4) | 135 (1.2) | 128 (1.7) | 122 (2.0) | 125 (1.6) | <0.001 | -20.2% | -2.0  (0.12) |
| ASE from LCS sources, g/d | 13 (1.3) | 14 (1.4) | 17  (1.0) | 15 (0.7) | 14 (0.9) | 13 (0.8) | 14 (0.8) | 12 (1.1) | 8.7 (0.8) | <0.001 | -32.3% | -0.25  (0.07) |
|  |  |  |  |  |  |  |  |  |  |  |  |  |
| **Children & adolescents: age2-19y** | |  |  |  |  |  |  |  |  |  |  |  |
|  |  |  |  |  |  |  |  |  |  |  |  |  |
| Total ASE, g/d | 165 (2.9) | 161 (3.2) | 153 (3.4) | 145 (2.3) | 143 (2.3) | 142 (1.8) | 129  (2.0) | 122 (2) | 125 (1.6) | <0.001 | -24.3% | -2.7  (0.16) |
| Total sugars, g/d | 162 (2.9) | 157 (2.8) | 149 (3.1) | 140 (2.4) | 138 (1.9) | 138 (1.7) | 125  (2.0) | 120 (2.2) | 123 (1.6) | <0.001 | -24.3% | -2.7  (0.16) |
| ASE from LCS sources, g/d | 3.0 (0.4) | 3.9 (0.6) | 4.3 (0.8) | 4.6 (0.6) | 5.3 (0.8) | 3.8 (0.4) | 3.5 (0.4) | 2.4 (0.4) | 2.3 (0.4) | 0.012 | -21.7% | -0.08  (0.03) |
|  |  |  |  |  |  |  |  |  |  |  |  |  |
| **Adults (age≥20y)** | |  |  |  |  |  |  |  |  |  |  |  |
|  |  |  |  |  |  |  |  |  |  |  |  |  |
| Total ASE, g/d | 172 (2.4) | 164 (3.2) | 158 (3) | 158 (4.2) | 151 (2.1) | 151 (1.7) | 147 (1.8) | 137 (2.7) | 137 (2.5) | <0.001 | -20.3% | -2.1  (0.17) |
| Total sugars, g/d | 155 (3.1) | 146 (2.3) | 136 (2.9) | 138 (3.7) | 134 (1.8) | 134 (1.6) | 129 (1.7) | 122 (2.3) | 126 (2.1) | <0.001 | -18.6% | -1.7  (0.16) |
| ASE from LCS sources, g/d | 17 (1.7) | 18 (2) | 21 (1.3) | 20 (1.1) | 17 (1.2) | 17 (1.1) | 18 (1.2) | 15 (1.5) | 11 (0.9) | <0.001 | -35.3% | -0.36  (0.09) |
|  |  |  |  |  |  |  |  |  |  |  |  |  |

^a^ Following the approach of described by Murakami & Linvingstone (Murakami and Livingstone 2015), potential under-reporters were identified as individuals for whom the ratio of reported energy intake to estimated basal metabolic rate (derived from Schofield’s sex- and age-specific equations using body weight) was less than 0.96. Approximately 25% of individuals were potential energy under-reporters. Individuals were excluded (n=891) from the under-reporting analysis if a valid objective weight measurement was not available. This table can be compared with **Table 2** from the main text to determine the impact of this exclusion of the observed trends.

**Supplemental Table 5.** Trends in approximate sugar equivalents (ASE) by population sub-group

|  | Fitted change from 2001-2 through 2017-2018 (95% CI) | | | | |  | |  |
| --- | --- | --- | --- | --- | --- | --- | --- | --- |
|  | Total ASE, g/d | P-interaction | Total sugars, g/d | P-interaction | ASE from LCS sources, g/d | | P-interaction | |
| Adults |  |  |  |  |  | |  | |
|  |  |  |  |  |  | |  | |
| Age group |  |  |  |  |  | |  | |
| 20-29 | -56.5  (-67.2, -45.8) | <0.001 | -52.8  (-63.0, -42.7) | <0.001 | -3.7  (-7.7, 0.4) | | <0.001 | |
| 30-39 | -47.1  (-57.2, -37.1) |  | -36.8  (-46.5, -27.1) |  | -10.4  (-15.5, -5.3) | |  |  |
| 40-49 | -36.7  (-46.9, -26.6) |  | -23.1  (-32.0, -14.1) |  | -13.6  (-19.3, -8.0) | |  |  |
| 50-59 | -15.4  (-28.0, -2.7) |  | -7.9  (-17.3, 1.5) |  | -7.5  (-13.8, -1.1) | |  |  |
| 60-69 | -7.9  (-16.9, 1.1) |  | -2.0  (-9.7, 5.7) |  | -5.8  (-10.3, -1.3) | |  |  |
| ≥70y | 2.7  (-3.3, 8.7) |  | -0.5  (-5.6, 4.7) |  | 3.1  (0.01, 6.3) | |  |  |
|  |  |  |  |  |  | |  | |
| Gender |  |  |  |  |  | |  | |
| Female | -26.0  (-31.0, -21.1) | 0.003 | -17.4  (-21.4, -13.3) | <0.001 | -8.6  (-11.8, -5.5) | | 0.03 | |
| Male | -38.4  (-45.5, -31.3) |  | -33.8  (-40.4, -27.1) |  | -4.6  (-7.8, -1.5) | |  |  |
|  |  |  |  |  |  | |  | |
| Race/ethnicity |  |  |  |  |  | |  | |
| Non-Hispanic White | -30.6  (-36.2, -25.0) | 0.99 | -25.2  (-30.1, -20.1) | 0.88 | -5.4  (-9.0, -1.8) | | 0.67 | |
| Non-Hispanic Black | -30.9  (-39.8, -22.0) |  | -27.3  (-36.2, -18.3) |  | -3.6  (-5.4, -1.8) | |  |  |
| Mexican-American | -31.2  (-38.8, -23.6) |  | -26.9  (-34.5, -19.4) |  | -4.3  (-6.6, -1.9) | |  |  |
|  |  |  |  |  |  | |  | |
| Family income to poverty ratio^a^ |  |  |  |  |  | |  | |
| <1.00 [lower income] | -20.3  (-31.3, -9.3) | <0.001 | -17.7  (-27.6, -7.8) | 0.001 | -2.6  (-5.7, 0.6) | | 0.007 | |
| 1-1.99 | -22.8  (-31.0, -14.5) |  | -19.5  (-27.2, -11.9) |  | -3.2  (-6.7, 0.3) | |  |  |
| 2-3.99 | -29.5  (-37.3, -21.6) |  | -23.1  (-30.6, -15.6) |  | -6.4  (-11.2, -1.6) | |  |  |
| ≥4.00 [higher income] | -43.2  (-49.9, -36.4) |  | -33.5  (-39.8, -27.1) |  | -9.7  (-14.6, -4.8) | |  |  |
|  |  |  |  |  |  | |  | |
| Education (age≥25y)^a^ |  |  |  |  |  | |  | |
| <HS | -10.1  (-19.3, -1.0) | <0.001 | -5.9  (-14.0, 2.3) | <0.001 | -4.3  (-8.3, -0.2) | | 0.065 | |
| HS | -29.7  (-39.4, -19.9) |  | -22.3  (-30.6, -13.9) |  | -7.4  (-12.2, -2.5) | |  |  |
| Some college | -27.0  (-34.8, -19.3) |  | -17.5  (-25.4, -9.7) |  | -9.5  (-14.1, -4.8) | |  |  |
| ≥College | -39.5  (-46.8, -32.2) |  | -29.5  (-36.9, -22.0) |  | -10.0  (-15.1, -5.0) | |  |  |
|  |  |  |  |  |  | |  | |
| Body mass index (kg/m^2^), adults^a^ |  |  |  |  |  | |  | |
| Healthy weight: 18-24.9 | -32.4  (-40.7, -24.2) | 0.44 | -27.5  (-36.1, -18.8) | 0.065 | -5.0  (-8.5, -1.4) | | 0.018 | |
| Overweight: 25-29.9 | -38.4  (-45.4, -31.4) |  | -29.8  (-36.3, -23.4) |  | -8.5  (-12.4, -4.7) | |  |  |
| Obese: ≥30 | -28.9  (-35.4, -22.4) |  | -18.2  (-24.2, -12.3) |  | -10.6  (-14.5, -6.8) | |  |  |
|  |  |  |  |  |  | |  | |
| Children & adolescents |  |  |  |  |  | |  | |
|  |  |  |  |  |  | |  | |
| Age group |  |  |  |  |  | |  | |
| 2-9y | -34.2  (-39.4, -28.9) | <0.001 | -33.3  (-38.2, -28.4) | <0.001 | -0.8  (-2.1, 0.4) | | 0.52 | |
| 10-19y | -52.6  (-59.2, -46.1) |  | -51.2  (-57.7, -44.7) |  | -1.4  (-2.7, -0.1) | |  |  |
|  |  |  |  |  |  | |  | |
| Gender |  |  |  |  |  | |  | |
| Female | -36.8  (-42.5, -31.0) | <0.001 | -35.5  (-40.9, -30.0) | <0.001 | -1.3  (-2.8, 0.2) | | 0.79 | |
| Male | -52.4  (-58.6, -46.3) |  | -51.4  (-57.3, -45.4) |  | -1.0  (-2.1, 0.04) | |  |  |
|  |  |  |  |  |  | |  | |
| Race/ethnicity |  |  |  |  |  | |  | |
| Non-Hispanic White | -44.4  (-51.5, -37.3) | 0.22 | -43.2  (-50.0, -36.4) | 0.29 | -1.2  (-2.8, 0.41) | | 0.37 | |
| Non-Hispanic Black | -38.1  (-45.5, -30.8) |  | -38.2  (-45.5, -30.9) |  | 0.04  (-0.8, 0.9) | |  |  |
| Mexican-American | -46.6  (-53.6, -39.6) |  | -46.2  (-53.1, -39.3) |  | -0.4  (-1.2, 0.4) | |  |  |
|  |  |  |  |  |  | |  | |
| Family income to poverty ratio^a^ |  |  |  |  |  | |  | |
| <1.00 [lower income] | -45.6  (-55.3, -35.7) | 0.81 | -45.5  (-55.0, -36.1) | 0.99 | 0.02  (-1.1, 1.1) | | 0.19 | |
| 1-1.99 | -39.6  (-49.1, -30.1) |  | -39.0  (-48.5, -29.5) |  | -0.6  (-2.0, 0.8) | |  |  |
| 2-3.99 | -44.4  (-52.6, -36.1) |  | -43.5  (-52.0, -35.0) |  | -0.8  (-2.2, 0.6) | |  |  |
| ≥4.00 [higher income] | -46.0  (-56.8, -35.2)_ |  | -43.9  (-54.0, -33.8) |  | -2.1  (-5.2, 1.1) | |  |  |
|  |  |  |  |  |  | |  | |
| Body mass index (kg/m^2^) |  |  |  |  |  | |  | |
| Underweight (<5%ile) | -41.4  (-53.8, -29.0) | 0.71 | -44.1  (-56.5, -31.7) | 0.97 | 2.7  (-0.06, 5.4) | | <0.001 | |
| Healthy weight (5-84.9%ile | -44.3  (-49.3, -39.3) |  | -43.7  (-48.5, -38.9) |  | -0.6  (-1.6, 0.4) | |  |  |
| Overweight (85-94.9%ile) | -44.5  (-55.3, -33.8) |  | -41.7  (-51.6, -31.7) |  | -2.9  (-5.5, -0.3) | |  |  |
| Obesity (≥95^th^ %ile) | -49.6  (-59.5, -39.7) |  | -44.8  (-54.4, -35.1) |  | -4.9  (-7.5, -2.2) | |  |  |

**References**

Dunford, E. K., D. R. Miles, S. W. Ng and B. Popkin (2020). "Types and Amounts of Nonnutritive Sweeteners Purchased by US Households: A Comparison of 2002 and 2018 Nielsen Homescan Purchases." Journal of the Academy of Nutrition and Dietetics **120**(10): 1662-1671.e1610.

Hootman, R. C. (1992). "Manual on descriptive analysis testing for sensory evaluation." Micro & Nano Letters.

Murakami, K. and M. B. Livingstone (2015). "Prevalence and characteristics of misreporting of energy intake in US adults: NHANES 2003-2012." Br J Nutr **114**(8): 1294-1303.
